# Fish DNA-modified clays: Towards highly flame retardant polymer nanocomposite with improved interfacial and mechanical performance

**DOI:** 10.1038/srep38194

**Published:** 2016-12-05

**Authors:** Omid Zabihi, Mojtaba Ahmadi, Hamid Khayyam, Minoo Naebe

**Affiliations:** 1 Institute for Frontier Materials, Deakin University, Geelong, Victoria, Australia; 2 Department of Chemical Engineering, Isfahan University of Technology, Isfahan, Iran

## Abstract

Deoxyribonucleic Acid (DNA) has been recently found to be an efficient renewable and environmentally-friendly flame retardant. In this work, for the first time, we have used waste DNA from fishing industry to modify clay structure in order to increase the clay interactions with epoxy resin and take benefit of its additional thermal property effect on thermo-physical properties of epoxy-clay nanocomposites. Intercalation of DNA within the clay layers was accomplished in a one-step approach confirmed by FT-IR, XPS, TGA, and XRD analyses, indicating that *d*-space of clay layers was expanded from ~1.2 nm for pristine clay to ~1.9 nm for clay modified with DNA (d-clay). Compared to epoxy nanocomposite containing 2.5%wt of Nanomer I.28E organoclay (m-clay), it was found that at 2.5%wt d-clay loading, significant enhancements of ~14%, ~6% and ~26% in tensile strength, tensile modulus, and fracture toughness of epoxy nanocomposite can be achieved, respectively. Effect of DNA as clay modifier on thermal performance of epoxy nanocomposite containing 2.5%wt d-clay was evaluated using TGA and cone calorimetry analysis, revealing significant decreases of ~4000 kJ/m^2^ and ~78 kW/m^2^ in total heat release and peak of heat release rate, respectively, in comparison to that containing 2.5%wt of m-clay.

Layered silicate clays have been widely utilized to equip the pristine polymers with value-added properties, such as considerable mechanical strength, thermal durability, and gas impermeability^1^,^2^. The final properties of polymer/clay systems dominantly depend on dispersion configuration of clay into polymer matrix and physico-chemical events at clay-polymer matrix interface^3^. Since the first attempt has been made to use layered silicate clays in construction of exfoliated nylon 6/clay nanocomposites by Toyota Company researchers^4^, several strategies have been developed to produce various exfoliated polymer/clay nanocomposites^5^. Nonetheless, due to the intense static forces among neighbouring platelets in the pristine layered silicate clays, complete exfoliation of platelets, and their further homogeneous dispersion in polymer matrices are still challenges to overcome^6^,^7^. Among various polymer-clay configurations, complete exfoliation of individual clay layers into the polymer matrices is of particular interest because it maximizes the interactions of clay layers with polymers matrix^8^. From the reactions kinetic viewpoint, to produce a completely exfoliated nanocomposite, theoretically, either polymerization reactions of monomers should be firstly initiated between clay galleries (which is so-called surface-initiated polymerization), and then is progressed to the bulk monomers, or polymerization rate between clay galleries should be faster than polymerization in bulk monomers, leading to the separation of clay layers^9^,^10^.

Layered silicate clay materials inherently have a hydrophilic nature and as such their compatibility with most industrial polymers is poor and consequently incorporation of unmodified clay into polymers, not only, does not improve performance of the polymers, but also potentially could deteriorate intrinsic properties of the parent polymers^11^,^12^. Most commercially available clays have been modified through cationic exchange process of clay interlayer cations with ammonium cations consisting of long alkyl hydrophobic chains, which lead to the expansion of clay layers and consequently facilitate the penetration of polymer chains into clay layers^13^,^14^. Although these modifications improve the dispersion of clay into polymer matrices, the interface of the modified clay layers with polymer matrices are not usually taken into account and the interactions at interface remain as weak as van der Waals interactions^15^,^16^ which could be accompanied with adverse plasticisation effects at clay-matrix interface^17^. Despite achievements in the exfoliation of clay layers into polymer matrices, it has been repeatedly reported that plasticisation has devastating effects on some mechanical properties of polymers, in particular glass transition temperature (*T*_g_) of epoxy polymers. To reduce the plasticisation effects, strong interactions e.g., covalent bondings between clay modifiers with polymer matrix are inevitably required to be established at interface^18^,^19^. In the case of epoxy polymers, it has been proven that the alkyl ammonium modifiers can catalyse self homo-polymerization of epoxide groups within clay layers, facilitating the exfoliation process^20^. Nevertheless, there is no interaction between clay layers and the formed epoxy matrix leading to the profound plasticisation effects on *T*_g_ of epoxy-clay nanocomposites. A common strategy to surmount the plasticisation effects at interface is coupling the hydroxyl groups of surface and edges of clay with polymer matrix through silane compounds^21^. The silane coupling agents can covalently react with polymer and reduce plasticisation effects^22^. Although it has been reported that clay treated with silanes can create a strong interface, a solvent process is required to achieve a highly individual layers dispersed into polymer matrix, which is not easily feasible in terms of its manufacturing^23^. Additionally, the amount of graftable hydroxyl groups on clay surface and edges are extremely limited and amount of silane grafted is low, in comparison to organic modifiers using cations exchange^24^. To overcome these challenges, modification based on cations exchange which appears to be more efficient in terms of its quantity, has to be formulated to create strong interactions between clay layers and polymer matrices, obtaning the desirable properties.

During the past decades, there has been a significant interest in use of sustainable and renewable materials instead of conventional hazardous substances for development of high-performance materials^25^,^26^,^27^. In this regard, a few approaches have been developed to modify layered silicate clays with natural compounds for polymer composites applications. Jin *et al*. coated montmorillonite with protein biopolymers extracted form soy plant using the pH change, leading to the exfoliation of clay layers into biopolymers^28^. Chitosan/clay nanocomposite is another example in which biopolymers are used to modify clay^29^. In the case of thermosetting epoxy/clay composites, Barua S. *et al*. reported a biocompatible epoxy/clay nanocomposite with enhanced mechanical properties for tissue engineering applications using modified bentonite with an oil derived from a specific plant^30^. Focusing on the role of interfacial physico-chemical interactions in mechanical properties of epoxy/clay nanocomposites, Yang L. *et al*.^31^ reported a biomimetic approach using *in situ* polymerization of dopamine within clay layers. In this approach, cationic amine groups of polydopamine were exchanged with clay cations, and the hydroxyl groups of polydopamine were tasked to enhance the interfacial interactions through forming hydrogen bondings with an epoxy polymer.

One of the most promising renewable materials, recently employed to enhance thermal performance of textile fabrics is DNA derived from fishing industrial waste^32^,^33^,^34^. It has been reported that DNA can act as an intrinsically flame retardant on cotton fabrics and enhance fire retardancy of system^32^,^35^,^36^,^37^. In contrast to the conventional fire retardant materials which are commonly phosphorous or halogen based hazardous compounds^38^,^39^, DNA is a green and natural flame suppressant and retardant which can potentially be replaced with traditional fire retardant materials^37^. General structure of DNA consists of sodium phosphate backbone groups, deoxyribose unites, and nucleobases having hydrogen bondings together. The sodium phosphates groups can potentially act as a nucleophile intermediate in organic reactions e.g., reaction with epoxide rings. Inspired by these features of DNA, we hypothesized that if DNA can be intercalated within clay layers, interfacial interactions as well as thermal performance of epoxy/clay system may significantly be improved in comparison to those commercially modified. To verify this hypothesis, we have embedded DNA within the clay layers and subsequently incorporated such DNA modified clay layers into an epoxy matrix to produce epoxy/clay nanocomposites. Herein, structure, morphology, mechanical, thermal, and flammability performance of these newly developed nanocomposites, have been comprehensively investigated while focusing on the role of interfacial interactions between modified clay and polymer matrix.

## Results and Disccusions

### DNA-modifed clay characterizations

**Figure 1:**
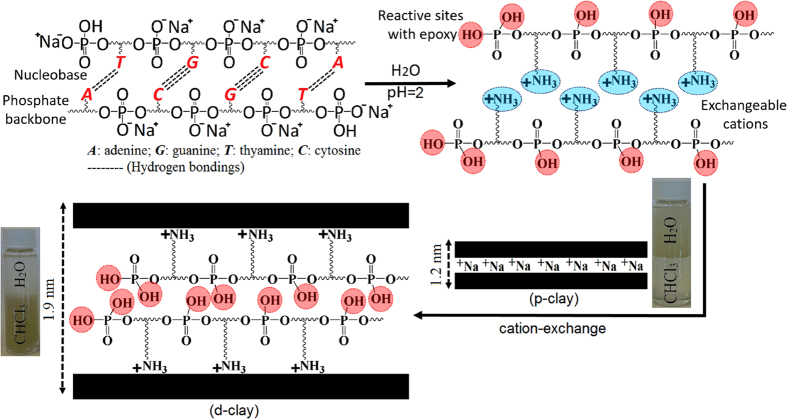
An ideal DNA structure and the chemical procedure to intercalate DNA within the clay and its solubility profiles.

**Figure 2:**
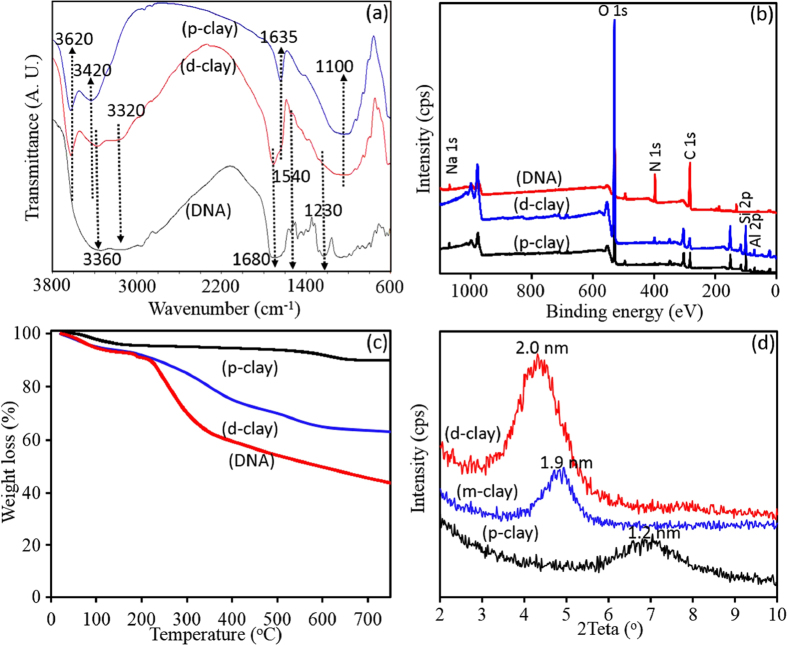
FTIR spectrums (**a**), XPS survey spectrum (**b**), TGA analysis (**c**), and XRD patterns (**d**) of the various samples.

Figure 1 demonstrates how to change DNA structures, being able to cation-exchange with clay cations, leading to intercalation of DNA within clay layers. Although dispersion of DNA/water makes a solution with pH ~5.5, it has been reported that hydrogen bonding between nucleobases of DNA structure could be effectively dissociated at pH ~4 causing to form ammonium cations through its nucleobases; and at pH < 2, DNA structure will be hydrolysed, causing to break the phosphodiester bonds and consequently the bases will be broken off^40^. As shown in Fig. S1, maximum amount of 72 ± 6 mg DNA per gram of p-clay was obtained to be intercalated within clay layers at pH = 2, and its amount decreases significantly at higher pHs. Ability to disperse the pristine clay (p-clay) and clay modified with DNA (d-clay) in solvents, are also presented in Fig. 1. As shown, d-clay becomes suspended into organic phase (chloroform) instead of being at water phase, whereas p-clay remains in water phase, which preliminarily confirms a transition of hydrophilicity nature of p-clay into the organophilicity in d-clay. The d-clay was fully characterized using FTIR, XPS, XRD, and TGA analysis to find out its structural characteristics. In FTIR spectrum of p-clay, both of the peaks at 3620 cm^−1^ and 3420 cm^−1^ are ascribed to H−O−H stretching vibration bands of water molecules bonded to the Si−O surface on the clay. The stretching bands of Al−OH and Fe−OH are also appeared at below 916 cm^−1^. The peak at 1635 cm^−1^ observed for p-clay can be attributed to the –OH deformation of water. The Si−O stretching vibration bands are observed around 1100 cm^−1^. After modification of clay with DNA, obvious new peaks at around 1230 cm^−1^, 1680 cm^−1^, and 3200 cm^−1^ were appeared in FTIR spectrums of d-clay and DNA, as indicated in Fig. 2a. These peaks are related to the P-O, P = O, primary/secondary N-H stretching, respectively, showing presence of DNA characteristic peaks in d-clay structure. While a broad peak related to the hydroxyl groups of both hydrogen phosphate groups and nucleobases can be observed after 3200 cm^−1^ in FTIR spectrum of DNA. Moreover, an obvious peak at 1450 cm^−1^ denotes presence of C = C stretching bonds in nucleobase of DNA structure for both d-clay and DNA samples. As shown in Fig. 2b, the main XPS characteristic peaks of p-clay are Si2p, Al2p, O1s and Na1s which appear at 103, 74, 533 and 1072 eV, respectively. After modification of p-clay with DNA, the main changes in XPS survey spectrum of d-clay are appearance of N1s and P2p as well as disappearance of Na1s, which clearly confirms successful modification. As the DNA structure includes sodium phosphate groups, appearance of P2p and disappearance of Na1s in XPS survey of d-clay shows that all Na^+^ cations were removed from d-clay structure. Table 1 also presents changes of surface elemental composition in atomic ratios relative to Al as its concentration remains constant. As it can be seen, ratios of C, N, and O to the Al increase significantly after DNA-based modification. Furthermore, Na/Al decreases to zero from 0.153, while P/Al increases from zero to 0.042 for d-clay in comparison to the p-clay. The difference of weight losses between d-clay and p-clay also confirms intercalation of DNA within clay layers. As presented in Fig. 2c, p-clay shows a minor weight loss related to de-absorption of moisture before 200 °C and another one after 600 °C, reaching total weight loss of ~8%. TGA thermograms of d-clay and DNA present a similar multi-step degradation and their total weight loss of d-clay and DNA reach ~37% and ~58%, respectively. The effect of intercalation of DNA within clay layer on basal *d*-spacing was investigated using XRD analysis. As shown in Fig. 2d, 2θ° decreases from ~7° for p-clay to ~4.9° for d-clay, corresponding to increasing the *d*-spacing from 1.2 nm for p-clay to 1.9 nm for d-clay. This increment in *d*-spacing of d-clay facilitates monomers penetration within clay layers. However, in comparison to the d-clay, m-clay has higher *d*-spacing which is due to having modifier based long alkyl chains.

Organophilicity of clays depends on wetting of modified clay by epoxy resin, which plays a significant role in dispersion quality in the matrix. The process of wetting of clays by epoxy resin consists of three types of wetting including adhesion wetting (*W*_*a*_), immersion wetting (*W*_*i*_), and spreading wetting (*W*_*s*_). The work of dispersion (*W*_*d*_) is the sum of these three aforementioned wetting terms which can be expressed as follows:


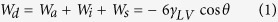


**Figure 3:**
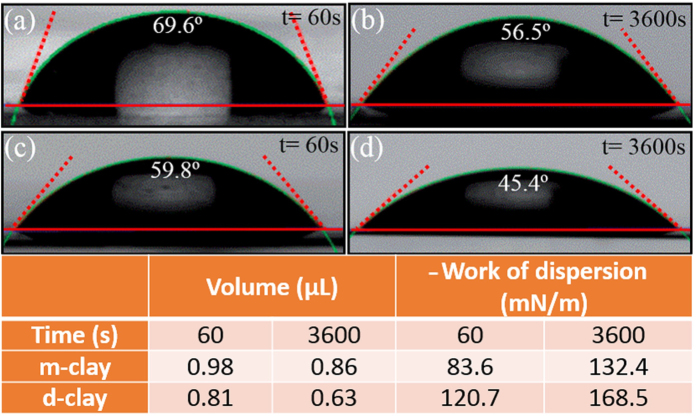
Evaluation of geometric shapes of epoxy droplet deposited on m-clay (**a**,**b**) and d-clay (**c**,**d**) at two different times, and their calculated work of dispersion and volumes.

Wetting and dispersion could be determined by the epoxy surface tension (*γ*_*LV*_) and contact angle between epoxy and nanoclay (θ°). *W*_*a*_, *W*_*i*_, and *W*_*s*_ are spontaneous when θ° < 90°^41^,^42^. Snap shots of epoxy droplet deposited on compacted discs of clay at different times duration (60 and 3600 seconds) are illustrated in Fig. 3. As shown, the angles formed between epoxy droplet and m-clay substrate are higher than that of d-clay. Aktas *et al*.^42^ declared that the contact angle of epoxy droplet on Cloisite 25 A nanoclay reaches a stable state of ~42° with a decrease of 16% in initial volume of epoxy drop. In comparison, angles formed between epoxy droplet with m-clay and d-clay reached ~69° and ~59°, respectively. However, higher decrease in initial volume of epoxy drop could be seen for both m-clay and d-clay. It is postulated that d-clay shows better affinity towards epoxy droplet. In other words, epoxy droplet could easily be absorbed to d-clay disk. Such phenomenon could be analyzed through volume changes in epoxy droplet observed on the samples. As presented in Fig. 3, a faster decrease in droplet volume with elapsed time proves that the penetration of epoxy droplet to d-clay is much higher than that of for m-clay. In other words, a decrease of ~84% in epoxy volume on d-clay was observed after 3600 s; however, its counterpart, m-clay shows a decrease of ~78% in epoxy volume. It is hypothesized that prompt impregnation of d-clay clusters due to higher wettability may imply a better dispersion quality and makes mechanical stirring and ultrasonication more effective. Moreover, the *W*_*d*_ could prove such observations. The highest *W*_*d*_ was observed for the d-clay. Compared with m-clay, a considerable increase ~27% could be observed for d-clay i.e., *W*_*d*_increases from 132.47 mN/m to 168.52 mN/m after 3600 s. This is arising from functional groups introduced to d-clay, being able to have physico-chemical interactions with epoxy resin, leading to strong repulsive forces to keep particles apart; consequently, the tendency to form agglomeration is supposed to be less in d-clay, compared to m-clay.

### Interfacial interactions

**Figure 4:**
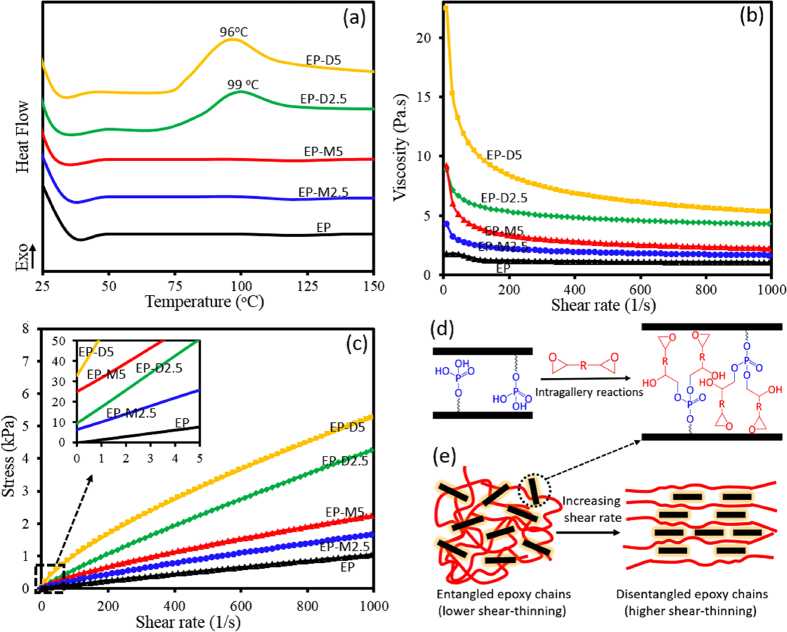
DSC thermograms (**a**), viscosity (**b**) and stress (**c**) versus shear rate for various un-cured epoxy suspensions; possible intra-gallery reactions in d-clay, and schematic presentation of shear-thinning behaviour of epoxy suspensions of containing d-clay.

Interfacial interactions between d-clay and m-clay with epoxy resin play a pivotal role in formation of different structures of epoxy-clay nanocomposites e.g., exfoliated/intercalated structures, which were studied by DSC and rheological analysis. Figure 4a depicts DSC thermograms of un-cured epoxy resin suspension containing various clays. As it can be seen, no curing reaction occurs during a dynamic heating of pure EP suspension and its nano-suspensions containing m-clays without hardener as evidenced by its thermogram which does not show any exothermic peak up to 150 °C, revealing that m-clays cannot have any effective interactions with the epoxy resin in this temperature range^43^,^44^. However, addition of 2.5 and 5 wt% d-clay in epoxy suspensions cause an exothermic peak to appear before 100 °C with enthalpies of −12.3 and −19.7 J/g, respectively. It is proposed that hydrogen phosphate groups intercalated between d-clay layers can react with the penetrated epoxy monomers into d-clay layers through ring opening of epoxide groups, schematically presented in Fig. 4d. These intra-gallery reactions could also facilitate diffusion of more epoxy monomers within the clay layers and are also responsible to expand the clay layers, inducing formation of exfoliated structures, before the extra-gallery reactions have been conducted by curing of the nanocomposites.

The interfacial interactions arising from these intra-gallery reactions were also explored by studying the changes of rheological behaviour of nano-suspensions. In this regard, viscosity and shear stress versus shear rate flow curves for nano-suspensions containing various clays are illustrated in Fig. 4b and c, respectively. The rheology behaviors of samples were analyzed by Herschel–Bulkley’s model according to the following equations:


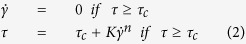


Where 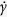 is the shear rate (s^−1^), *τ* and *τ*_*c*_ are, respectively, the shear stress and yield stress. The *K* and *n* are the flow consistency index and the flow index, respectively. Flow index determines the flow behavior. In other words, *n* < 1 for shear thinning behavior and *n* > 1 for shear thickening behavior could be observed in the nano-suspensions. The Herschel–Bulkley’s model parameters were calculated and presented in Table 2. Epoxy nanocomposites containing 2.5 and 5%wt of d-clay and m-clay are named as EP-D2.5 and EP-D5, and EP-M2.5 and EP-M5, respectively.

As illustrated in Fig. 4b and c and presented in Table 2, it is argued that addition of d-clay not only could increase the viscosity of epoxy resin but also promote shear-thinning behavior, steaming from interfacial interactions due to the intra-gallery reactions. In other words, compared with m-clay, DNA as a reactive modifier could physico-chemically involve and entangle with the epoxy chains, leading to a higher viscosity which could induce yield stresses in nano-suspensions. Compared with nanosuspensions containing 2.5 wt% m-clay, an increase of ~19 Pa in τ_c_ is observed for suspensions reinforced with the same content of d-clay. Another extra reason behind such trend could be related to the temporary formation of hydrogen bonding between hydroxyl resulting in initial resistance toward shear stress with functional groups of DNA carbohydrates. Such phenomenon is more obvious at high contents of d-clay. To put it differently, compared with nanosuspension filled with 5 wt% m-clay, the addition of the same content of d-clay to epoxy shows an increase of ~23 Pa in τ_c_. It could be deduced that role of DNA as reactive modifier in increment of viscosity as well as *τ*_c_ would be more effective in higher contents because interfacial interactions lead to decrease the possibility of agglomeration formation, which causes more d-clay to be involved in formation of network. Moreover, the same increasing trend is observed for flow consistency, whereas a decreasing trend could be detected for flow index.

As viscosity behavior of the nano-suspensions also depends on nanoclay dispersion levels into epoxy matrix, another prerequisite condition for viscosity discussion is the relation of dispersion level with flow index. As discussed in literatures^45^,^46^, it was investigated that lower values of the flow index imply higher levels of uniform dispersion of nanoclay into polymer matrix. Therefore, compared with m-clay, d-clay is prone to be more-uniformly dispersed in epoxy system. It is assumed that dispersion of d-clays into epoxy suspensions could lead to delaminated structures by increasing the d-spacing of d-clay layers, resulting from intra-gallery reactions. Therefore, it is postulated that each individual platelet could efficiently restrict the mobility of epoxy chains, on the one hand, and promote shear thinning behavior, on the other hand. As presented in Table 2, the lowest values of *n* e.g., 0.76 and 0.72 are observed for the nano-suspensions containing 2.5 and 5 wt% d-clay, respectively, whereas dispersion of m-clays into epoxy resin could not induce the same shear-thinning performance. In other words, higher shear-thinning could be only seen when clay is modified with DNA based modifier. It is argued that although modification of clay with DNA could increase viscosity of nano-suspensions, we subscribe to the view that a significant decrease in the entanglement density of epoxy molecules could be obtained. It could imply that aligned d-clay could act like slippery agents, schematically presented in Fig. 4e. This phenomenon is in agreement with the observation reported in the literatures^47^,^48^. As clay concentration increases, the viscosity of epoxy resin increases, which is mostly accompanied by inducing heterogeneity in the system. Such heterogeneity is arising from agglomerations and micro-voids formed in epoxy resin while processing^49^. It is worth to consider that increasing the m-clay content into epoxy suspension from 2.5 wt% to 5 wt% makes the intercalation/exfoliation more and more difficult. As a result, the weakest shear-thinning tendency could be seen for nano-suspension containing 5 wt% m-clay. This means that it leads to a low alignment of clay layers, which causes nanosuspension to resist more against higher shear rates.

### Nanocomposites structure

**Figure 5:**
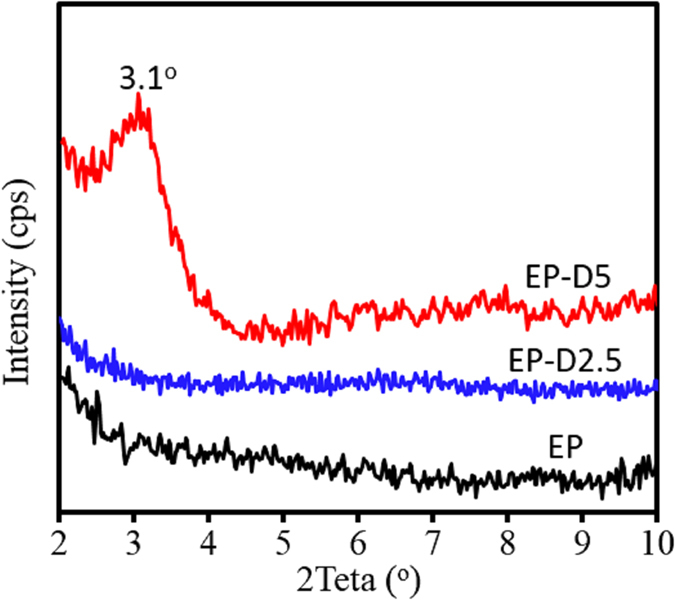
XRD patterns of pure EP and its nanocomposites.

In order to verify the hypothetical considerations discussed in DSC and rheological analyses, nano/micro-structures of epoxy nanocomposites containing d-clay were examined by XRD and TEM analysis. Figure 5 demonstrates XRD patterns of pure EP and epoxy nanocomposites containing d-clays. As shown, there is no peak in the XRD pattern of pure EP in the 2θ° of 2°–10°, showing an amorphous structure for epoxy matrix. Therefore, if a XRD peak appears in this region for the nanocomposites, it should be related to the clay structure and its basal *d*-spacing in the nanocomposite. As can be seen for EP-D2.5 sample, this nanocomposite system shows no peak in its XRD pattern, demonstrating that initial *d*-spacing of dry d-clay which was ~1.9 nm, is completely expanded so that its 2θ° becomes <2° (equaled to *d*-spacing of >4.4 nm). This finding reveals the present of exfoliated structures in this nanocomposite. In contrast, EP-D5 system has an obvious XRD peak reflection in 2θ° = 3.1° which implies formation of the induced intercalated clay structures into epoxy nanocomposite at higher d-clay content with a basal *d*-spacing of ~2.8 nm.

**Figure 6:**
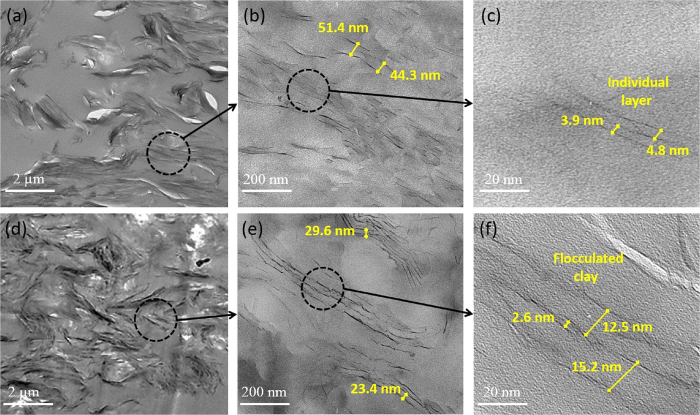
TEM micrographs of EP-D2.5 (**a**–**c**), and EP-D5 (**d**–**f**) nanocomposites.

Details of nanocomposites structure were investigated by TEM observations as illustrated in Fig. 6. As depicted in Fig. 6a and d, the dark lines in these figures are related to the silicate nanolayers and the light sections are related to epoxy matrix. It was mentioned that penetrated epoxy monomers within semi-separated clays treated by DNA modifier, could enhance clay layers separation through intra-gallery reactions and consequently such phenomenon induce some semi-stacked clays to be partially exfoliated, as it can be seen from TEM of EP-D2.5 nanocomposite (Fig. 6b). Furthermore, the intercalated structures could also be observed for this nanocomposite. It is argued that incorporation of higher contents of clay could merely result in interacted structures. As shown in Fig. 6e, three individual intercalated ordered structures so-called “intercalated tactoids”, could be detected for EP-D5 nanocomposite. However, compared with the EP-D5, EP-D2.5 possesses thin tactoids containing only a few clay layers. These small tactoids are uniformly and randomly dispersed in the epoxy resin, demonstrating that the clay modification based DNA is an effective approach to enhance both the exfoliation and dispersion of clay. In addition to such argument associated with dispersion, as reported in literatures^50^,^51^,^52^, under an effective load, most of microcracks are initiated within the intra-layer of semi-stacked clay rather than at epoxy-clay interfacial region. This phenomenon proves that higher contents of d-clay, e.g., 5 wt%, could result in lower reinforcing trend in tensile strength mentioned in mechanical section. In other words, in comparison with low d-clay content (Fig. 6c) which contains individual layers of clay, high content of d-clay (Fig. 6f) could result in flocculated structures. As shown in Fig. 6c, an exfoliated configuration having individual layers was formed, further confirming by absence of a reflection peak in XRD pattern of EP-D2.5. Moreover, although phase-separated clay tactoid structures is observable in Fig. 6f, such morphology implies that tendency of d-clay to agglomeration at high content outweighs complete delamination of clay layers due to low penetration of epoxy monomers into stacked layers of modified clay^53^. To conclude, more homogenous distribution, alongside well dispersion, could be observed for EP-D2.5, in comparison with EP-D5.

### Mechanical and thermo-mechanical performance

**Figure 7:**
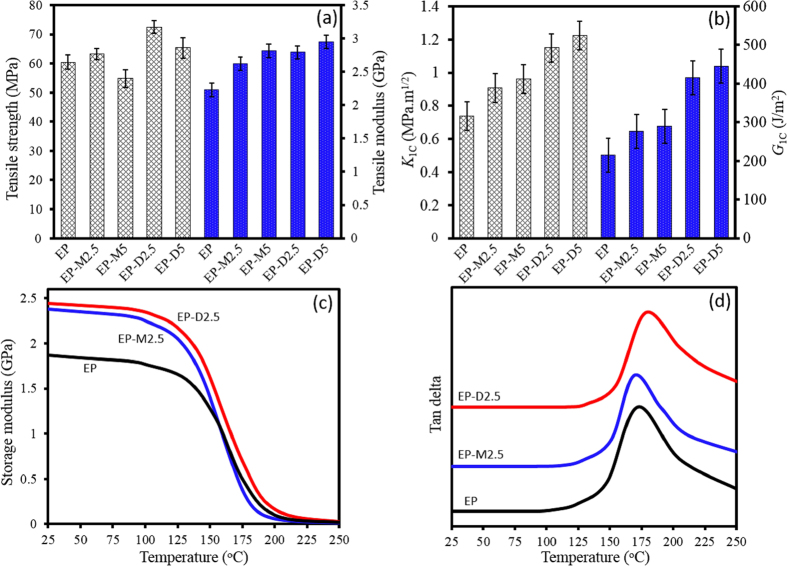
Tensile (**a**) and fracture toughness properties (**b**), and storage modulus (**c**) and tan δ (**d**) vs temperature for pure EP and its various epoxy nanocomposites.

The mechanical properties of epoxy nanocomposites containing various concentrations of m-clay and d-clay are shown in Fig. 7a and b. As it can be seen, the addition of m-clay has not improved the tensile strengths of epoxy matrix significantly and in fact the addition of 2.5 wt% of m-clay to epoxy resin (EP-M2.5) resulted in only ~5% increase in tensile strength, compared to pure EP. While, addition of 5 wt% of m-clay to epoxy matrix (EP-M5) not only did not increase the tensile strength but also led to a ~9% decrease. However, the inclusions of 2.5 and 5 wt% of d-clay in epoxy resin (EP-D2.5 and EP-D5) resulted in ~20% and ~8% increase in tensile strength of epoxy composites, respectively. This could be due to the improved dispersion of nanoclay as well as stronger filler-matrix physico-chemical interactions achieved through DNA modification of nanoclay. These interactions not only improve the epoxy monomer diffusion through faster intra-gallery reaction but also react and entangle with epoxy chains. This reinforcing mechanism will lead to the promoted strengths in epoxy nanocomposites.

On the contrary, as evidenced by DSC and rheological analysis presented earlier, m-clays do not interact effectively and covalently with epoxy resin compared to d-clays. When it comes to moduli, it is argued that the moduli of nanocomposites could be improved by adding either m-clay or d-clay. It means that although interfacial adhesion could enhance nanocomposite properties, moduli are mostly controlled by some factors such as: (i) high aspect ratio of a single clay platelet, (ii) higher stiffness of fillers, and (iii) restriction of polymer chain mobility^11^,^19^. As illustrated in Fig. 7a, it is worthy to mention that nanocomposites containing d-clay still show higher moduli than those containing m-clay. This is possibly arising from higher exfoliation degree of d-clay in epoxy matrix, resulting in higher stress-transferring and shear deformation mechanism. Mostly, fracture toughness and critical strain energy release rate of epoxy systems reinforced with nanoclay have been improved through various mechanisms such as pull-out, bridging effect, and interface debonding being observed in morphology section^50^. Compared with EP-M systems, EP-D systems exhibit higher toughness which is due to the fact that d-clay layers, adhering perfectly to epoxy resin through covalent bonding, are capable of carrying and transferring the highest amount of stress applied to matrix, resulting in higher absorbent of fracture energy. As presented in Fig. 7b, fracture toughness of EP-D2.5 and EP-D5 increased by ~56% and ~66%, respectively, compared to the EP. Whereas, the inclusion of the 2.5 and 5 wt% of m-clay could lead to ~23 and ~30% increases in fracture toughness. The same trend could be also observed for critical strain energy release rate. According to earlier observations made by Miyagawa *et al*.^54^ and Le Pluart *et al*.^55^, it has been hypothesized that higher intercalation degree of nanoclay might deflect crack more efficiently than exfoliated platelets due to the vulnerability to fracture. This leads to higher fracture toughness of EP-D5 in comparison to EP-D2.5, as TEM and XRD results of EP-D5 showed higher intercalation degree of d-clay into epoxy matrix compared to the EP-D2.5. Therefore, such enhancements confirm reinforcing potential of d-clay in high-performance epoxy nanocomposites, providing better stress-transfer. For comparison, Zaman *et al*.^11^ reported that the addition of 2.5 wt% of clay treated by various reactive modifiers with different chains length having free amine-end groups result in ~36%, ~18%, and ~8% decrease in tensile strengths and ~21%, ~44%, and ~58% increases in fracture toughness of epoxy nanocomposites. It is believed that the length of surfactant molecule and its ability to react with matrix could affect mechanical properties. According to Wang *et al*.^27^, using a green approach in preparation of nanocomposites, the highest improvement of ~22% in tensile strength could be achieved for the epoxy systems reinforced with 1 wt% of Cloisite30B.

Figure 7c illustrates the DMTA plots of storage modulus (*E*’) versus temperature for various epoxy systems. Moreover, as presented in Fig. 7d, the tan*δ*, which is the ratio of the loss modulus to the storage modulus, gives insight into polymer chains movement in relation with the strength of the epoxy system. From temperature corresponding to the maximum value of tan*δ*, glass transition temperature (*T*_g_) can be obtained. Moreover, crosslink density of the epoxy systems could be evaluated using following equation^56^,^57^,^58^:


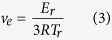


Where *v*_e_ is the estimation of crosslink density, and *R* is the universal gas constant. *E*_r_ is storage modulus corresponding to the *T*_r_ where *T*_r_ is *T*_g_ + 30, and *E*_g_ is also defined as storage modulus corresponding to the *T*_g_−30.

As it can be seen from Fig. 7c and data presented in Table 3, addition of 2.5%wt clay regardless of its modification increases storage modulus at *T* < *T*_g_ which is glassy region of epoxy system. However, the storage modulus of EP-M2.5 system become lower than that of the EP at *T* > *T*_g_. In other words, the EP has higher storage modulus at its rubbery region in comparison to EP-M2.5. This is because of the formation of plasticity effect on epoxy matrix at the interface with m-clay, leading to a reduction in ability of load transfer from matrix to the m-clay, causing lower *T*_g_ of EP-M2.5 in comparison to the EP^17^. As presented, *T*_g_ of the EP decreases from 172 °C to 169 °C when incorporating m-clay into epoxy matrix, which is in agreement with other reports^22^,^59^,^60^. In contrast, storage modulus of EP-D2.5 is higher that of both pure EP and EP-M2.5 in both glassy and rubbery regions at all temperatures. However, this higher storage modulus is more conspicuous at glassy region, in comparison to the rubbery region. The *T*_g_ of EP-D2.5 shows 6 °C and 9 °C increases, respectively compared to the pure EP and EP-M2.5. These increments not only do denote that no plasticity effect at interface of d-clay with epoxy is present, but also show that d-clay can establish a strong interface with epoxy, leading to the restriction of segmental chains motion. Moreover, the EP-D2.5 shows 0.35 mmol/m^3^ increment in the crosslink density while a significant reduction was observed for the crosslink density of EP-M2.5, compared to the EP system. This means that delaminated/exfoliated d-clay layers provide a higher surface available to be encountered with epoxy matrix, being able to have chemical bondings with the matrix, whereas a high crosslink density at interface of m-clay with epoxy matrix is effectively hindered by inducing the plasticity effect.

**Figure 8:**
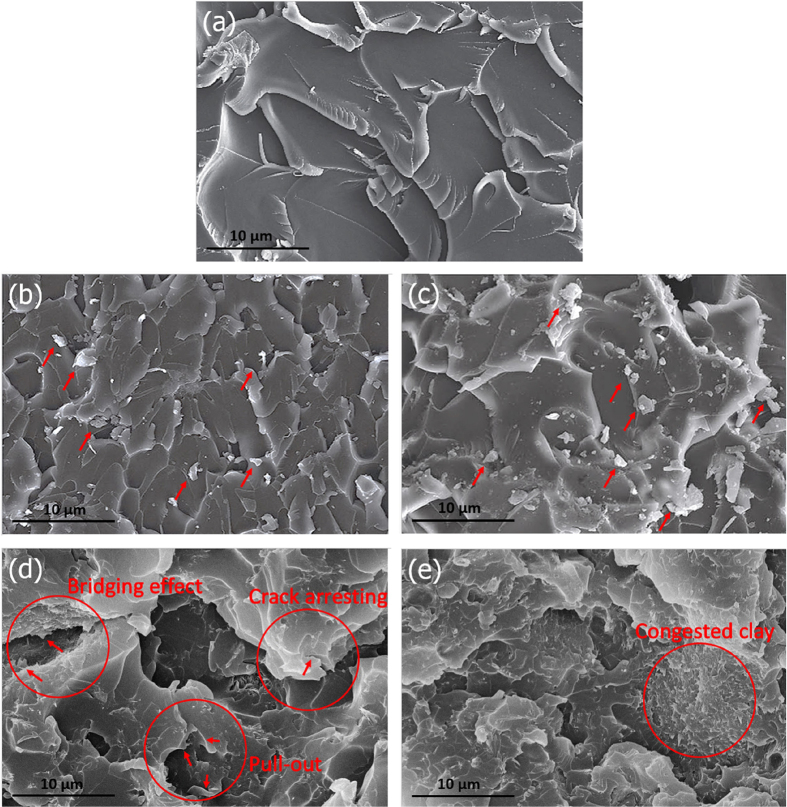
Fracture surfaces of pure EP (**a**), EP-M2.5 (**b**), EP-M5 (**c**), EP-D2.5 (**d**), and EP-D5 (**e**) nanocomposites.

Figure 8 shows fracture surfaces of the EP and its various nanocomposites. Although surface morphology of the PE is mostly smooth, it is possible to observe some approximately large fracture surfaces, as shown in Fig. 8a. On the contrary, when m-clay and d-clay are added into epoxy matrix, the crack propagates through matrix tortuously resulting in smaller fracture plates (Fig. 8b–e). This type of fracture is arising from crack deviation while applying load^61^. Moreover, compared with EP-M systems, the effect of d-clay incorporated into epoxy matrix (EP-D systems) on crack growth resistance via different mechanisms such as crack arrest, birding effect, and pull-out is more tangible (Fig. 8d). This phenomenon could be explained by effective interfacial interactions and homogenous dispersion, achieved by DNA modified clay. On the other hand, as m-clay does not have an effective adhesion to matrix as discussed above, the rejected m-clays from epoxy matrix are simply observable. Such occurrence causes reduction of mechanical performance, as discussed in pervious sections. Another consideration related to these reinforced epoxy composites is related to the poor levels of dispersion and micro-void formation at higher contents of nanoclay. In other words, the addition of higher contents of clay (e.g., 5 wt%) could result in agglomeration formation, causing lower filler/epoxy surface interactions. Although EP-D5 system possesses a few inevitable agglomerations, its morphology exhibits mostly disorderly congested d-clay (Fig. 8e). This means that it is highly likely that DNA modification could cause clay not only to be well-separated but also to be presented at least in congested forms instead of agglomerations. At higher loading of d-clay, it is hypothesized that they are prone to get closer. This could possibly lead to the formation of accumulation of intercalated clay instead of highly exfoliated one. Generally, as crack encounters nanoclay platelet, different scenarios can be assumed due to the micron-sized lateral dimension. The crack could bypass nanoclay platelets either by breaking them or pulling them out from matrix, as illustrated in Fig. 8d. In both conditions, the crack energy will be dissipated^62^. Therefore, when nanoclay is modified, the matrix could hold it tightly and restrict it from being easily pulled out. As a result, compared with m-clay, d-clay possessing strong interfacial bonding with matrix consumes crack propagation energy more and more. Another point related to taking advantage of DNA modified clay is that the possibility of interlayer delamination of clay under mechanical loading could decrease. In other words, the intercalated clay with enough *d*-spacing could lead to epoxy monomer diffusion. As epoxy monomer is diffused, elastic force applied by epoxy molecules cross-linking inside the clay galleries leads to exfoliation of clay layers i.e. swelling of clay galleries occurs^49^,^63^. Additionally, the ability of DNA modifier to react with epoxy through chemical bonding keeps clay layers to be firmly embedded within the matrix while load is applied. This will result in a more effective stress-transfer mechanism^64^. In contrast, despite the fact that epoxy monomer can also diffuse into stacked m-clay layers due to its initial *d*-spacing resulting from long alkyl chain quaternary ammoniums, its interactions with epoxy molecules still remains as weak as van der Waals forces, which can act like flaws in composites causing their premature failures and delamination, under mechanical loadings. Moreover, the higher chance of formation of agglomerates in m-clay could intensify such devastating effects and as such the properties of such composites will be similar to the micro-particle filled composites^49^.

### Thermal and flammability performance

**Figure 9:**
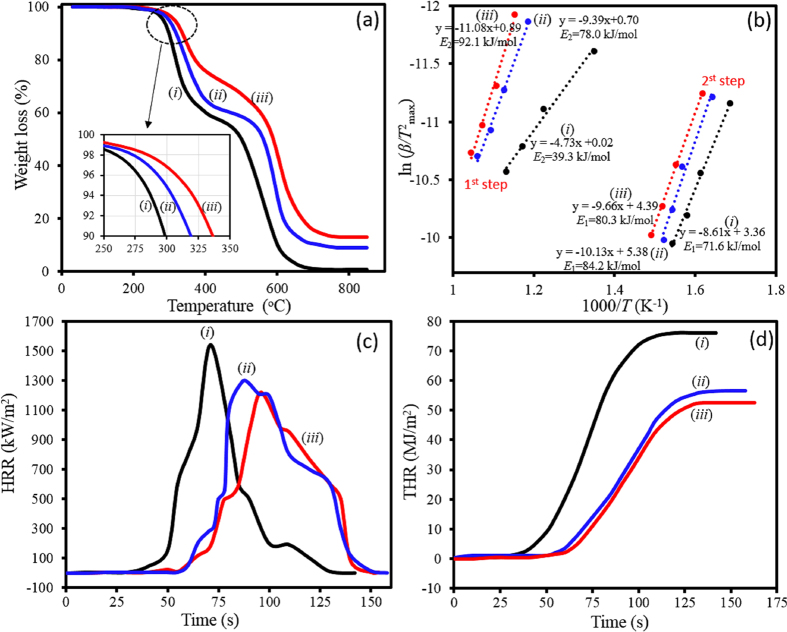
TGA thermograms (**a**), Kissinger plots for thermo-oxidative degradation (**b**), HRR vs time (**c**), and THR vs time (**d**) for the pure EP (i), EP-M2.5 (ii), and EP-D2.5 (iii) systems.

We have investigated the effect of DNA as clay modifier and natural flame retardant on the thermal properties of epoxy-clay nanocomposites. In order to have a comprehensive evaluation, thermal performance of nanocomposites was examined by TGA and cone calorimetry to compare the thermo-oxidative degradation and flammability properties. Figure S2 and Fig. 9a show TGA thermograms of various epoxy nanocomposites at different heating rates under the air flow and the results are presented in Table S1 and Table 4. As it can be seen, thermo-oxidative behaviour of epoxy nanocomposites shows a multi-step degradation consisting of two main steps. Herein, we considered various parameters including *T*_i_ and *T*_max_ (temperatures corresponding to 5% weight loss and maximum degradation rate for each step, respectively), char yield at 850 °C, and total activation energy required for thermo-oxidative degradation (*E*_1_ + *E*_2_ = *E*_total_), in evaluation of thermal properties using TGA analysis. Activation energy for each degradation step was calculated using Kissinger method^65^, as it is independent of any presumption on the degradation mechanism according to the following equation:


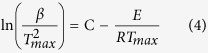


Where *β* is heating rate, and *C* is constant. By plotting 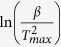 vs 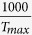 and fitting a straight line, the *E* value can be obtained using the slope, which their plots are presented in Fig. 9b.

As expected, addition of 2.5%wt clay regardless of its modifier type enhances all thermal characteristics of epoxy matrix. Regarding the effect of DNA modifier on the thermal properties, 16 °C, 6 °C, and 19 °C increases in *T*_i_, *T*_max,1_, and *T*_max,2_ are observed, respectively, for nanocomposite incorporated with 2.5 wt% d-clay when compared to that incorporated with 2.5 wt% m-clay. As shown in Table 4, char yield of the EP after thermo-oxidative process at 850 °C is insignificant (0.75%). The char yield increased upon incorporation of clay and the increase was more profound for EP-D2.5, compared to EP/M2.5. Moreover, *E*_total_ of EP-D2.5 is ~172 kJ/mol which is ~10 kJ/mol higher than that of the EP-M2.5. These TGA results show a significant additional thermal stability effect on epoxy nanocomposites containing d-clay, resulting from DNA intercalated within clay layers.

Combustion behaviours of samples were also examined by cone calorimetry as a useful tool in evaluating the flame retardancy performance under a forced-flaming combustion. Figure 9c and d display plots of heat release rate (HRR) and total heat release (THR) versus time, respectively. Using these plots, various parameters including THR, peak of heat release rate (PHRR), and time of reaching peak of heat release rate (*t*_PHRR_) were extracted and summarized in Table 4.

**Figure 10:**
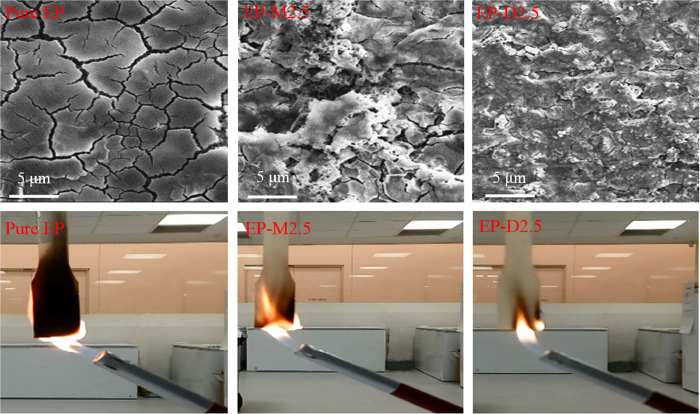
SEM images of char residues after cone calorimetry tests, and flame test photographs in the 10th second for the various samples.

The results demonstrate that pure EP exhibits a high PHRR of 1542 kW/m^2^. The PHRR value of EP-M2.5 is 1298 kW/m^2^ which is only 224 kW/m^2^ lower than that of pure sample. While d-clay exhibits a notable flame retardant effect on epoxy system and the addition of 2.5%wt d-clay into the epoxy matrix results in a 322 kW/m^2^ reduction in PHRR. Moreover, *t*_PHRR_ of pure EP increases from 71 s to 87 s and 96 s for the EP-M2.5 and EP-D2.5, respectively. This shows that EP/D2.5 requires 9 s longer to reach its PHRR, in comparison to EP/M2.5. The mechanism behind this observation stems from intrinsically flame retardancy of DNA modifier, possessing phosphate groups which can act as a barrier in formation of char. Moreover, DNA can release ammoniac and carbon dioxide gas under heating conditions and reduce flammability of the system^33^. It was also observed that THRs of both nanocomposites show significant differences in comparison to the pure EP. However, THR of EP-D2.5 is ~4000 kJ/m^2^ lower than that of the EP-M2.5. These remarkable reductions in PHRR and *t*_PHRR_ values of EP-D2.5 could also result from the insulation barrier effect of a cohesive and compact char layers on postponing the oxygen diffusion and the escape of volatile decomposition compounds produced during the combustion^2^,^66^. This fact was further investigated by SEM observations on the char residues structures. As illustrated in Fig. 10, the char residues of the pure EP show a rickety surface having wide cracks. Although EP-M2.5 surface exhibits lower cracks in comparison to pure EP, it still has an incompact surface. On the other hand, EP-D2.5 shows a dense and fully compacted surface morphology without any cracks on its surface, leading to the lower efficiency of heat and volatiles transfer due to the obstructing effect, and consequently providing underlying epoxy matrix with an effective barrier^67^. Fire propagation was simply evaluated through the keeping a flame near the samples, which their photographs are presented in Fig. 10. As it can be clearly seen, the fire quickly propagates across the pure EP sample; while it encounters a delay for the nanocomposites. Moreover, an obvious slower fire propagation are observed for the EP-D2.5 in comparison to the EP-M2.5, confirming an additional fire resistivity effect of DNA-modified clay on flammability of epoxy polymer.

We have compared the PHRR and THR values as well as mechanical performance of the epoxy-clay nanocomposite containing 2.5 wt% d-clay with the published reports in literatures in which epoxy nanocomposites contain both low and high nanofiller loadings. As shown in Table S2, a considerable low amount of d-clay can bring about acceptable figures in terms of improvements in both mechanical and flammability properties. This is while in other published reports mostly high loadings of nanofiller has led to only improvements in flammability performance. Such deduction can be proved by comparing our results with results reported in literature. According to the data presented in Table S2, two different trends can be observed for comparison. The first trend is dealing with the case when the amount of nanofiller is approximately as same as the amount of d-clay e.g. 2.5 wt%. In this condition, the reported decrements in PHRR and THR are significantly lower than that of d-clay we are reporting herein. Considering the second trend, it can be said that more decreases in PHRR and THR can be seen for epoxy nanocomposites containing high amount of nano fillers which are usually destructive in terms of mechanical performance. In other words, at high nanofiller loadings, low mechanical performances are expected to be observed in various mechanical properties including tensile strength, tensile modulus, and fracture toughness. Mostly, the reduction in mechanical properties is attributed to poor dispersion and weak interfacial adhesion. However, in this study, through DNA modification, the aim is to improve mechanical performance and provide clay with reinforcing features through chemical interactions. The study presented here, demonstrates a balance between mechanical and flame properties. In other words, compared to other modified clays presented in current literatures, a small loading of DNA modified clay shows a great potential to enhance flame retardancy of epoxy composites while improving its mechanical performance.

Moreover, in order to evaluate the contribution of DNA in overall flame retardency of the epoxy systems, PHRR, THR, and *t*_PHRR_ values of epoxy composites containing neat clay, neat fish DNA were also obtained and their results are presented in Table S3. As it can be seen, addition of 2.5 wt% neat clay to epoxy (EP-N2.5 sample) can lead to insignificant decreases of ~4.6% and ~8.5% in PHRR and THR, respectively. Whereas the addition of the same amount of m-clay (EP-M2.5 sample) results in ~15.8% and ~25.7% reduction in PHRR and THR, respectively. Moreover, the PHRR and THP decrease by ~20.8% and ~31.2% for the epoxy nanocomposite containing 2.5 wt% d-clay (EP-D2.5) which demonstrates the highest flammability improvement in epoxy resin studied herein. This reveals the improved dispersion and barrier effect of clay as a result of DNA modification. In addition to this, two different amounts of neat DNA powder (0.2 and 2.5 wt%) are solely incorporated into the epoxy matrix to evaluate the effect of DNA agent on the flammability properties. It is worth to mention that 0.2 wt% DNA was calculated as maximum amount of DNA grafted within clay layers which obtained at pH = 2, as discussed in Fig. S1. Therefore, 0.2 wt% DNA was incorporated into epoxy matrix to compare with other epoxy systems. The results of flammability study of these samples show that the reduction in PHRR and THR are slightly lower than that of EP-M2.5 composite because the amount of DNA (0.2%) was much lower than that of m-clay (2.5 wt%). However, when the same amount of neat DNA powder (2.5 wt%) was used to produce the epoxy composite (EP**-**DNA2.5 sample), the highest improvement in both PHRR and THR were observed which are ~60.3% and ~51.1% decreases in PHRR and THR, respectively. This clearly confirms that DNA is an effective agent for improvement of flame retardency of epoxy polymer systems.

Nonetheless, despite the role of DNA in improving the flammability performance of clay-epoxy nanocomposites, DNA to a great extent is incompatible and unprocessable with epoxy resin only which extremely hinder fabrication of epoxy-DNA composites with appropriate mechanical performance. Therefore, to take advantages of DNA performance, it should be grafted on a proper platform such as clay prior to incorporating into epoxy to provide composites with both improved mechanical and flame properties. In other words, both DNA and clay are set to cooperate with each other considerably through various mechanisms: (i) DNA can contribute to uniform clay dispersion as well as clay-matrix interactions leading to greater mechanical and flame properties, (ii) DNA agent can yield further improvement in flame properties via its functional groups having phosphorous and nitrogen, (iii) the reduction in mechanical properties which may be caused by DNA agent can be compensated by well dispersed clay. As a result, both flame and mechanical properties can be enhanced.

## Conclusions

Waste DNA from fishing industry has a great potential for recovery and re-use as a source of flame retardant materials in nanocomposites while improving strength. Herein, for the first time we have shown that fish DNA can be used in modification of clay nanomaterials for preparation of epoxy nanocomposites with significantly improved mechanical and flammability properties. Based on the results obtained in this study, the following detailed conclusions can be drawn for the proposed application:

- The results of epoxy droplet contact angle revealed ~44% increase in work of dispersion which is a critical factor in determination of epoxy resin compatibility and reactivity, and ~17% further decrease in penetration of epoxy droplet into DNA modified (d-clay), both in comparison to a commercially modified clay e.g., Nanomer I.28E.
- The dispersion levels of d-clay into epoxy matrix studied by XRD and TEM analyses confirmed the outstanding role of DNA as a modifier and its remarkable influence on the well dispersed structures including intercalated/exfoliated structures arising from intra-gallery polymerization.
- The rheological behaviours of epoxy-clay nanosuspensions, as another evidence for dispersion and interaction, proved the possibility of interactions between d-clay and epoxy monomers leading to formation of a network, which possesses high viscosity level and being resistance to shear rates. It was concluded that d-clay attached to epoxy chains could act as slippery agents to promote shear thinning behaviour.
- Inclusion of d-clay into epoxy resin led to a significant improvement in tensile strengths, moduli and fracture toughness compared to composites containing m-clay. This phenomenon results from improved clay-matrix interfacial adhesion, better dispersion and more effective role of d-clay in consumption of crack energy through various mechanisms such as crack arresting, deviation, and pull out procedures as confirmed by SEM micrographs. Observation of ~3% increase in *T*_g_ of epoxy/d-clay system versus ~1% decrease for epoxy/m-clay system, both compared to pure epoxy system, demonstrates that plasticity effect of nano-clays on *T*_g_ of epoxy nanocomposite was eliminated as a result of the effective interfacial interactions.
- Contribution of DNA molecules to the considerable improvement of thermal stability and fire resistancy of epoxy-clay systems was approved by TGA and cone calorimetry results. This improvement is as a result of the formation of condensed char layers during combustion due to the release of effective suppressant agents during the decomposition of DNA structures.

## Methods

### Materials

Epoxy resin (diglycidyl ether of bisphenol A, D.E.R 332) and diethylenetriamine as curing agent were obtained from Sigma-Aldrich and used as received. The used pristine clay was sodium montmorillonite and the organoclay was a commercial product under the name of Nanomer I.28E, which were supplied by Nanocor Co., USA. DNA powder from herring sperm was supplied from Sigma-Aldrich and stored at below 8 °C. All the solvents used in this study were of analytical grade.

### Intercalation of DNA within clay layers (d-clay)

To intercalate the DNA structures into clay layers, as-received DNA (2.00 gr) was dispersed into 200 ml DI water by stirring for 1 h, followed by adding 1.0 M HCl aqueous solution to adjust the pH to 2, 3, 4, and 5. The resultant solutions were stirred for further 3 h at 60 °C. In a separate beaker, pristine clay (2.00 g) was dispersed into 200 ml boiling water and stirred for 2 h before sonicated for 1 h in an ultrasonic bath. The dissolved and pH adjusted DNA solutions were added to the clay/DI water suspension and further stirred for 6 h to allow for the complete cation exchange process. The final mixtures were then filtered and washed several times with abundant DI water until no chloride detected by adding 0.1 N AgNO_3_ solution. The obtained DNA-modified clays (d-clays) at various pHs were then vacuum dried at 60 °C prior to use. By measuring differences between initial pristine clay weight with various d-clays weight, amount of intercalated DNA at each pH can be calculated, which is presented in Fig. S1. It is found that the highest intercalation of DNA on pristine clay occurs at pH = 2.

### Epoxy-clay nanocomposites preparation

**Figure 11:**
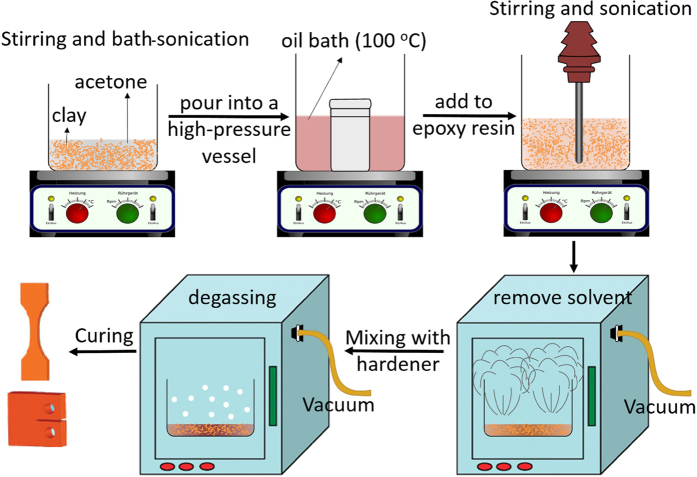
Schematic showing the dispersion of clay in the epoxy matrix via the simplified “slurry-compounding” process.

To take the full advantage of solvent properties in increasing the layers spacing in clay, fabrication of polymer nanocomposites were conducted according to the “slurry-compounding” process^22^, but with major modifications to simplify it and to assure that the clay concentration does not change during the process. As illustrated in Fig. 11, in a typical experiment, 1.00 g d-clay obtained at pH = 2 or Nanomer I.28E (m-clay) were dispersed in 100 ml acetone and stirred for 2 h, followed by sonication using an ultrasonic bath for 1 h to form a fine slurry before pouring the slurry into a high-pressure vessel and heating up to 100 °C for 12 h. This process facilitates the penetration of acetone between clay layers. After cooling to room temperature, proper amounts of epoxy resin were added to the clay/acetone slurry and stirred at 70 °C for 6 h. The mixture then was sonicated, for 30 min using a Hielscher UIP1000-230 ultrasonic processor operating at a frequency of 15 kHz to generate ultrasonic waves with an amplitude of 80 μm peak-to-peak through the epoxy suspensions with an ultrasonic pulsing cycle of 2 s on and 2 s off, being kept in an ice bath. To completely remove the acetone, the epoxy mixtures were subjected to the vacuum at 60 °C for 24 h. Then, a stoichiometric amount of hardener was added to the compositions before applying the vacuum for 30 min to degasify the bubbles produced during mixing the hardener. The total mixtures were poured into a mould and finally the curing process was conducted at 70 °C for 6 h, followed at 120 °C for 2 h. A pure epoxy sample was also prepared using the same condition and considered as control sample and named pure EP sample. Epoxy nanocomposites containing 2.5 and 5%wt of d-clay and m-clay were named EP-D2.5 and EP-D5, and EP-M2.5 and EP-M5, respectively. For flammability comparisons, the epoxy systems containing 2.5% and 0.2% neat DNA powder as well as 2.5% neat clay were also prepared using the above-mentioned procedure, named EP-DNA2.5, EP-DNA0.2%, and EP-N2.5%, respectively.

### Characterizations

FT-IR spectra were recorded with KBr pellets containing the samples on a FTIR spectrophotometer of Bruker Optics. X-ray photoelectron spectroscopy (XPS) analysis was performed using an AXIS Nova spectrometer (Kratos Analytical Inc., Manchester, UK) with a monochromated Al K_α_ source at a power of 180 W (15 kV × 12 mA) and a hemispherical analyser operating in the fixed analyser transmission mode. Survey spectra were acquired at a pass energy of 160 eV. The atomic concentrations of the detected elements were calculated using integral peak intensities and the sensitivity factors supplied by the manufacturer. XRD patterns were obtained using a PANalytical X’Pert Pro Diffractometer with Cu K*α* radiation (λ = 1.54184 Å), operated in 2°–10° (2*ϑ°*) at 45 kV and 30 mA with a step size of 0.033. The spreading of an epoxy droplet on compacted discs of clay, provided with a compaction pressure of 20 MPa, was analysed using KSV Model CAM101 Contact Angle Meter (KSV Instruments Ltd, Finland) equipped with an Olympus DP70 high resolution microscope at ambient temperature. A 4 μL droplet of epoxy was poured onto compacted discs with diameter and thickness of 13 mm and 4 mm, respectively; and the amount of epoxy droplet penetrated to each clay substrate was evaluated by digital image analyser.

DSC analyses were performed using a TA Q200 DSC instrument in high purity nitrogen atmosphere. The samples were heated up to 150 °C at the heating rate of 10 °C/min. From the exotherms obtained, the heat of reaction and the peak temperature were determined. Rheological evaluations were carried out using a TA DHR 3 rheometer with cone–plate geometry. A cone with a diameter of 40 mm and a tilt angle of 2° were utilized, and gap width was fixed to be 49 μm. The range of shear rate, used in this experiment, was chosen to be between 0–1000 1/s. The nanosuspensions were located between the cone and plate and soaked for five minutes. Dynamic mechanical properties of the epoxy-clay nanocomposites were examined using a TA Instruments Q800 in the cantilever bending mode. The instrument was calibrated before use and the samples were prepared according to ASTM E1640 before being mounted on a single cantilever clamp. The DMA analysis were carried out from 25 °C to 250 °C at a heating rate of 2 °C/min and the frequency value of 1 Hz. TGA tests of various modified clay were carried out using a Perkin–Elmer TGA instrument at the heating rate of 10 °C/min under a steady nitrogen flow of 60 ml/min. While, TGA analyses of polymer nanocomposites were operated at various heating rates under an air flow of 100 ml/min. Flammability of the polymer nanocomposites were examined by cone calorimeter (Fire Testing Technology, UK) and measurements were performed at an incident heat flux of 35 kW/m^2^, according to the ISO5660 standard.

The fracture surfaces of tensile samples were examined using a scanning electron microscope (SEM) operated at 25 kV. The fracture surfaces were gold-coated prior to microscopy observations. Transmission electron microscope (TEM) samples with specimens of approximately 80 nm in thickness were prepared using a Leica Ultracut UCT ultramicrotome at room temperature. Microtomed sections were imaged by a Philips TEM at 300 kV in bright field mode. Tensile tests were performed on dog-bone samples according to ASTM D638 Type I by using an Instron universal testing machine; cross-head speed 5 mm/min with a 30 kN load cell. Moreover, according to ASTM D 5045, fracture toughness was measured using the compact tension specimen (see Fig. S3) with dimensions of 48 mm × 48 mm width × 10 mm at 10 mm/min. An instantly propagating crack was designed for each specimen by tapping a razor blade to the samples because as mentioned in literature^11^ it is the most economical approach to create a satisfactory sharp crack. To obtain statistically meaningful results, the tensile properties and fracture toughness of at least five specimens for each case were averaged and reported. Fracture toughness properties were shown as mode-l stress intensity factor (K_1C_) and critical strain energy release rate (G_1C_) according to following equations:


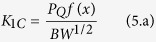



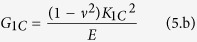



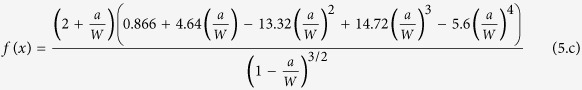


And 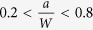


Where *P*_Q_, *B, W, a, E*, and *ν* are the maximum load, the thickness, the width, crack length, Young’s modulus, and Poisson’s ratio, respectively. According to literatures, Poisson’s ratio is considered 0.35 for DER 332 epoxy resin^68^.

## Additional Information

**How to cite this article**: Zabihi, O. *et al*. Fish DNA-modified clays: Towards highly flame retardant polymer nanocomposite with improved interfacial and mechanical performance. *Sci. Rep.* **6**, 38194; doi: 10.1038/srep38194 (2016).

**Publisher's note:** Springer Nature remains neutral with regard to jurisdictional claims in published maps and institutional affiliations.

## Supplementary Material

Supplementary Information

## Figures and Tables

**Table 1:** Surface elemental composition of the p-clay and d-clay in atomic ratios relative to aluminium taken as a unique elemental marker for clay structure.

Samples	Al/Al	C/Al	O/Al	N/Al	P/Al	Na/Al
p-clay	1.000	1.632	8.884	0.108	0.000	0.153
d-clay	1.000	1.854	9.605	0.474	0.042	0.000

**Table 2:** Herschel–Bulkley’s model parameters obtained from rheology behavior of various nano-suspensions.

Suspension	Yield stress τ_c_ (Pa)	Flow consistency *K* (Pa.s^n^)	Flow index *n*
Pure EP	0	1.81	0.92
EP-M2.5	6.52	5.35	0.83
EP-M5	9.47	10.63	0.87
EP-D2.5	25.22	11.14	0.76
EP-D5	33.02	35.82	0.72

**Table 3:** Results of DMTA analyses for cured epoxy systems.

Sample	EP	EP-M2.5	EP-D2.5
*T*_g_ (°C)	172	169	178
*E*_r_ (MPa)	99	80	120
*E*_*g*_ (GPa)	1.43	1.81	1.92
*v*_e_ (mmol/m^3^)	1.96	1.61	2.31

**Table 4:** Thermal characteristics of various nanocomposites obtained by TGA and cone calorimetry analyses.

System	*T*_i_ (°C)	*T*_max,1_ (°C)	*T*_max,2_ (^o^C)	%Char yield at 850 °C	*E*_total_ (kJ/mol)	PHRR (kW/m^2^)	*t*_PHRR_ (s)	THR (MJ/m^2^)
Pure epoxy	283	320	468	0.75	110.9	1542	71	76.2
EP-M2.5	299	339	572	9.2	162.2	1298	87	56.6
EP-D2.5	315	345	591	13.3	172.4	1220	96	52.4
