# Supplementary material for: Fish DNA-modified clays: Towards highly flame retardant polymer nanocomposite with improved interfacial and mechanical performance
Source: Sci Rep. 2016 Dec 5;6:38194. doi: 10.1038/srep38194 (PMC5137040; doi:10.1038/srep38194)
Supplement: Supplementary Information [file srep38194-s1.pdf]

Supplementary Information for:

Fish DNA-modified clays: Towards highly flame retardant polymer nanocomposite with improved interfacial and mechanical performance

Omid Zabihi[1], Mojtaba Ahmadi[2], Hamid Khayyam[1], Minoo Naebe[1*]
[1]Institute for Frontier Materials, Deakin University, Geelong, Victoria, Australia
[2]Department of Chemical Engineering, Isfahan University of Technology, Isfahan, Iran.

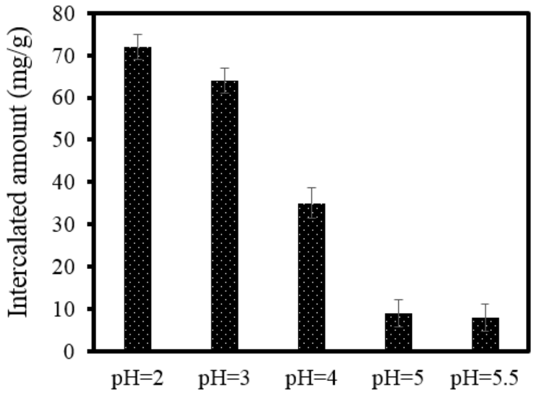

Fig S1. The amount of interacted DNA within clay layers at various pHs.

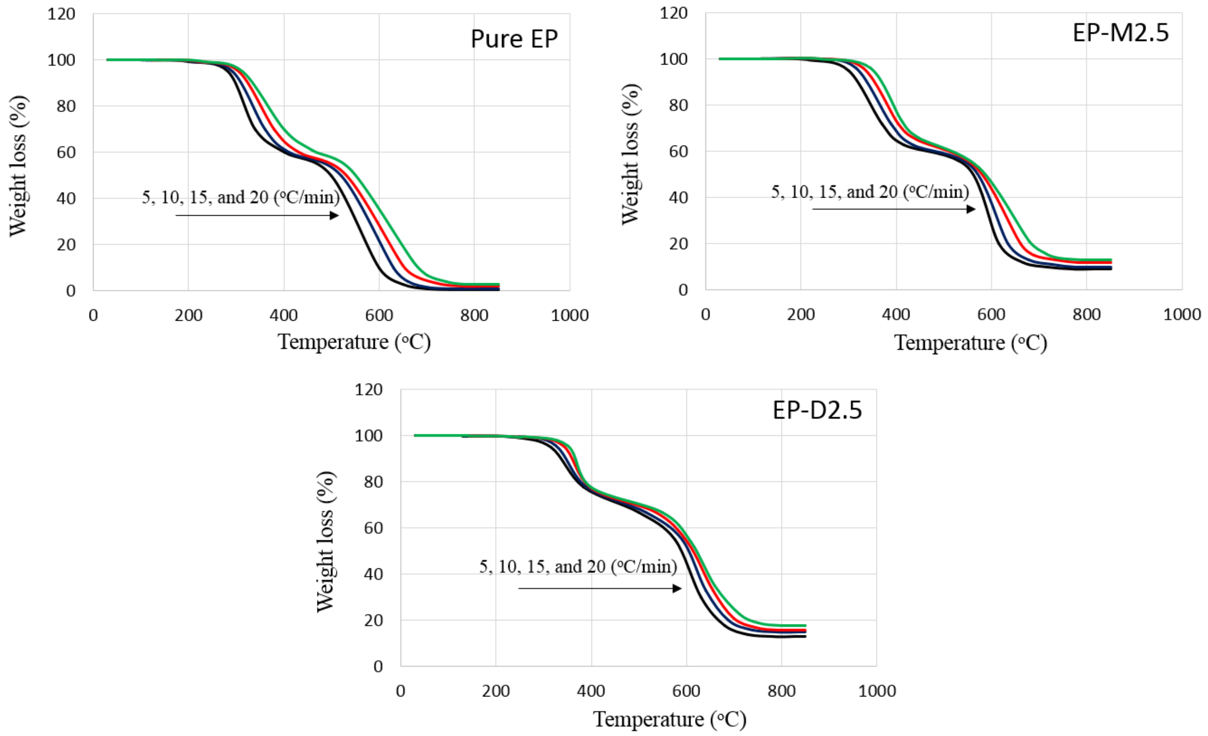

Fig. S2. TGA thermograms of pure epoxy and its nanocomposites under an air flow at various heating rates.

---

*Corresponding author: Tel: +61403667708

E-mail: minoo.naebe@deakin.edu.au

Table S1. Thermal characteristics of pure epoxy and its nanocomposites during thermo-oxidative degradation process.

Pure EP				
**Heating rate (°C/min)**	**T_i (°C)**	**$T_{max,1}$ (°C)**	**$T_{max,2}$ (°C)**	**Char yield at 850 °C (%wt)**
5	283	320	468	0.75
10	297	347	544	1.2
15	305	360	580	2.3
20	314	375	610	3.5
**EP/M2.5**				
**Heating rate (°C/min)**	**T_i (°C)**	**$T_{max,1}$ (°C)**	**$T_{max,2}$ (°C)**	**Char yield at 850 °C (%wt)**
5	299	336	571	9.2
10	309	365	615	10.4
15	316	375	642	12.8
20	322	384	669	14.1
**EP/D2.5**				
**Heating rate (°C/min)**	**T_i (°C)**	**$T_{max,1}$ (°C)**	**$T_{max,2}$ (°C)**	**Char yield at 850 °C (%wt)**
5	315	345	591	13.3
10	322	371	633	15.2
15	331	385	662	16.5
20	342	398	686	18.8

Table S2. Summary of the effect of various modified nanomaterials on the flammability and mechanical properties of epoxy composites as reported in the literature.

Type of modified nano material	*Content in epoxy matrix (wt. %)*	*Peak of heat release rate (%)*	*Total heat release (%)*	*Fracture Toughness (%)*	*Critical strain energy release rate (%)*	*Tensile strength (%)*	*Tensile Modulus (%)*	*Ref No.*
**DNA-clay**	**2.5**	**-20.88**	**-31.23**	**+56**	**+93.1**	**+20**	**+25.11**	**This study**
POSS-MMT[a]	2	-4.28	-9.65	------	------	------	------	1
Fullerenes modified clay	3	-5.34	-6.52	------	------	------	------	2
$BaTiO_3$–PANI	15.38[b]	-37.57	-18.57	------	------	+233	+20	3
octadecyl ammonium montmorillonite	10	-38	------	------	------	------	------	4
Molybdenum disulphide wrapped carbon nanotube	2	-18.4	-31.26	------	------	------	------	5
Graphene nanosheets	3	+5.08	-15.56	------	------	------	------	6
Graphene functionalized with phosphorous compounds	5	-35	------	------	------	------	------	7
Polyaniline-stabilized silica nanoparticles	1	-6.14	-0.37	------	------	+23.12	+12.10	8
	5[c]	-14.94	-4.86			+13.66	+37.50	
Clay modified with phosphorous compounds (ED2-2) [d]	4	-54.4	-48.91	------	------	------	------	9
Sepiolite clay – dehydrated	2	-13.02	-0.61	------	------	------	------	10
Sepiolite clay – hydrated	2	+5.54	+3.38					
Alkyl quaternary ammonium modified clay	5	+5.34[e]	-7.14[e]	------	------	------	------	11
		-4.35[f]	0[f]					
Vinyl triphenyl phosphonium bromide clay	5	-25.15[e]	-12.5[e]					
		-6.66[f]	-9.09[f]					
Octadecyl ammonium ion-modified clay	5	-13.48[e]	0[e]					
		-12.09[f]	+2.59[f]					
Double walled carbon nanotubes	0.5	-11.66[e]	-5.36[e]					
		-3[f]	-10.39[f]					
eth-clay [g]	2.5	------	------	+21.13	+36.25	-36.56	+7.72	12
m27-clay [g]				+44.26	+89.88	-17.81	+9.62	
xtj-clay [g]				+58.19	+114.91	-7.81	+16.45	

[a] octaammonium polyhedral oligomeric silsesquioxane-modified montmorillonite.
[b] contains 5.38 wt% barium titanate (BaTiO3) and 10 wt% conductive polyaniline (PANI).
[c] Doped with H3PO4.
[d] ED2-x indicates that the x means the weight of organoclay in epoxy which is modified with 2 wt. % of 9,10-dihydro-9-oxa-10-phosphaphenanthrene-10-oxide (DOPO).
[e] MY721 as an epoxy resin and a curing agent 4,40-diaminodiphenyl sulphone (Aradur 976-1) were used.
[f] Araldite LY5052 as an epoxy resin and a hardener based on modified cycloaliphatic amines (HY5052) were used.
[g] Ethanolamine (eth); JEFFAMINE M2070 monoamine (m27); XTJ502 (JEFFAMINE ED-2003) were used as different modifiers for clay.

Table S3. Flammability characteristics of various epoxy systems obtained by cone calorimetry analyses.

Sample	*PHRR* *(kW/m²)*	*THR* *(MJ/m²)*	t_{PHRR} *(s)*
Pure epoxy	1542	76.2	71
EP-N2.5[a]	1471	69.7	76
EP-M2.5[b]	1298	56.6	87
EP-D2.5[c]	1220	52.4	96
EP-DNA0.2[d]	1343	60.8	83
EP-DNA2.5[e]	611	37.2	120

[a] Epoxy resin containing 2.5 wt. % of neat clay.
[b] Epoxy resin containing 2.5 wt. % of nanomer I.28E organoclay (m-clay).
[c] Epoxy resin containing 2.5 wt. % of DNA modified clay (d-clay).
[d] Epoxy resin containing 0.2 wt. % of neat DNA powder without clay.
[e] Epoxy resin containing 2.5 wt. % of neat DNA powder without clay.

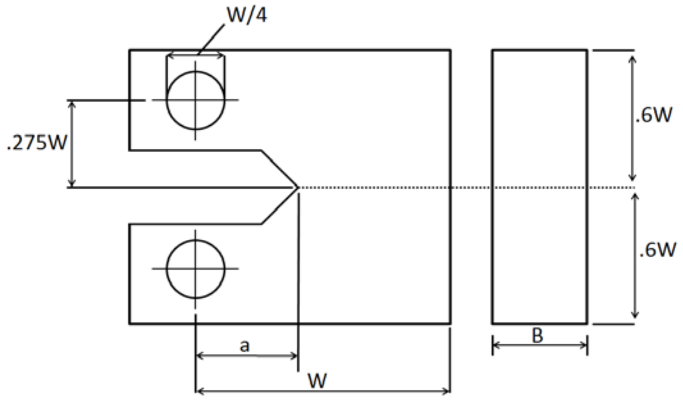

Fig S3. Schematic presentation of dimensions of a CT Specimen.

References:

1 Pan, M. *et al.* Morphology, thermal properties, and fire behavior of epoxy resin nanocomposites containing octaammonium polyhedral oligomeric silsesquioxane-modified montmorillonite. *High Performance Polymers*, doi:10.1177/0954008313493101 (2013).

2 Tsai, T.-Y., Bunekar, N., Huang, C.-C., Huang, Y.-S. & Chen, L.-C. Novolac cured epoxy resin/fullerene modified clay composites: applied to copper clad laminates. *RSC Advances* **5**, 95649-95656, doi:10.1039/C5RA18073B (2015).

3 Zhang, X., He, Q., Gu, H., Wei, S. & Guo, Z. Polyaniline stabilized barium titanate nanoparticles reinforced epoxy nanocomposites with high dielectric permittivity and reduced flammability. *Journal of Materials Chemistry C* **1**, 2886-2899, doi:10.1039/C3TC30129J (2013).

4 Camino, G., Tartaglione, G., Frache, A., Manferti, C. & Costa, G. Thermal and combustion behaviour of layered silicate–epoxy nanocomposites. *Polymer Degradation and Stability* **90**, 354-362, doi:http://dx.doi.org/10.1016/j.polymdegradstab.2005.02.022 (2005).

5 Zhou, K. *et al.* MoS2 Nanolayers Grown on Carbon Nanotubes: An Advanced Reinforcement for Epoxy Composites. *ACS Applied Materials & Interfaces* **7**, 6070-6081, doi:10.1021/acsami.5b00762 (2015).

6 Liu, S., Yan, H., Fang, Z. & Wang, H. Effect of graphene nanosheets on morphology, thermal stability and flame retardancy of epoxy resin. *Composites Science and Technology* **90**, 40-47, doi:http://dx.doi.org/10.1016/j.compscitech.2013.10.012 (2014).

7 Qian, X. *et al.* Novel organic-inorganic flame retardants containing exfoliated graphene: preparation and their performance on the flame retardancy of epoxy resins. *Journal of Materials Chemistry A* **1**, 6822-6830, doi:10.1039/C3TA10416H (2013).

8 Gu, H. *et al.* Flame-Retardant Epoxy Resin Nanocomposites Reinforced with Polyaniline-Stabilized Silica Nanoparticles. *Industrial & Engineering Chemistry Research* **52**, 7718-7728, doi:10.1021/ie400275n (2013).

9 Weng, Z., Senthil, T., Zhuo, D., Song, L. & Wu, L. Flame retardancy and thermal properties of organoclay and phosphorous compound synergistically modified epoxy resin. *Journal of Applied Polymer Science* **133**, n/a-n/a, doi:10.1002/app.43367 (2016).

10 Zotti, A. *et al.* Effects of sepiolite clay on degradation and fire behaviour of a bisphenol A-based epoxy. *Composites Part B: Engineering* **73**, 139-148, doi:http://dx.doi.org/10.1016/j.compositesb.2014.12.019 (2015).

11 Katsoulis, C., Kandare, E. & Kandola, B. K. The effect of nanoparticles on structural morphology, thermal and flammability properties of two epoxy resins with different functionalities. *Polymer Degradation and Stability* **96**, 529-540, doi:http://dx.doi.org/10.1016/j.polymdegradstab.2011.01.002 (2011).

12 Zaman, I. *et al.* Interface-tuned epoxy/clay nanocomposites. *Polymer* **52**, 497-504, doi:http://dx.doi.org/10.1016/j.polymer.2010.12.007 (2011).
